# Marital Status, Social Integration, and Suicidal Thoughts and Behaviors in the Military Health and Well‐Being Project

**DOI:** 10.1002/jclp.70093

**Published:** 2026-01-20

**Authors:** Katherine Musacchio Schafer, Sean P. Dougherty, Marie Campione, Ruth Melia, Emma Wilson‐Lemoine, Thomas Joiner

**Affiliations:** ^1^ Department of Biomedical Informatics Vanderbilt University Medical Center Nashville Tennessee USA; ^2^ Department of Psychiatry and Behavioral Sciences Vanderbilt University Medical Center Nashville Tennessee USA; ^3^ Orlando VA Medical Center Orlando Florida USA; ^4^ Florida State University Tallahassee Florida USA; ^5^ University of Limerick Limerick Ireland; ^6^ Kings College London London UK

**Keywords:** marital status, social integration, suicidality, veterans

## Abstract

Suicidal thoughts and behaviors (STBs) are a public health concern, particularly among Veterans, who experience elevated rates of STBs. Social integration is negatively associated with STBs, such that high social integration is correlated with low rates of STBs. Much of the literature has studied marital status as a social relationship that may protect individuals from STBs. Although largely unstudied, it has long been assumed that social integration moderates the link between marital status and STBs. Thus, in a sample of Veterans (*N* = 1469; Military Health and Well‐Being Project), we tested our hypotheses that (1) social integration varies by marital status (single vs. married vs. domestic partnership vs. divorced vs. widowed), (2) social integration is negatively associated with STBs (i.e., lifetime suicidal ideation, past year suicidal ideation, suicidal ideation communication, and likelihood of suicide attempt), (3) STBs vary based on marital status, and (4) social integration moderates the cross‐sectional link between marital status and STBs. Results indicated that (1) marital status was associated with social integration, such that married people reported greater social integration than people who were single; (2) social integration was negatively associated with all four STBs (*r's* < − 0.09, *p* < 0.001); (3) STBs varied based on marital status such that married people reported fewer STBs than people who were in a domestic partnership, single, widowed, or divorced, and (4) social integration did not moderate the link between marital status and STBs. Findings indicate that factors external to marital status may contribute to the link between social integration and STBs.

## 1 Introduction

Prevention of suicidal thoughts and behaviors (STBs) is a national priority (Centers for Disease Control and Prevention, 2023). Every year more than 48,000 Americans die by suicide (American Foundation for Suicide Prevention, 2023). Despite increased study of suicide (Schafer et al. 2021a) and increased U.S. government funding of suicide prevention efforts (whitehouse.gov), suicide rates have risen significantly over the last 20 years. Military Veterans are at particular risk, exhibiting elevated rates of STBs as compared to members of the general population who did not serve in the military (VA.gov). In 2020, the unadjusted suicide rate among Veterans was nearly double that as compared to non‐Veteran U.S. adults (i.e., 31.7 per 100,000 vs. 16.1 per 100,000; VA.gov).

### 1.1 Theories of Suicide

Researchers have long proposed theories to understand the causes of STBs. Previous work (Schafer et al. 2020; 2021b) reviews of common theories of suicide, including the Interpersonal Theory (Van Orden et al. 2010), Motivational‐Volitional Theory (O'Connor and Kirtley 2018), Three‐Step Theory (Klonsky and May 2015), and Hopelessness Theory (Beck et al. 1975). Each of the former three theories is couched within the Ideation‐to‐Action framework and proposes that reduced social integration, whether conceptualized as thwarted belongingness, loneliness, loss of connection, or lack of connectedness, contributes to the development of suicidal ideation. In these theories, social integration is articulated as how readily people reach out, count on, and depend on other people in their network. Thus, when people lose or have no one to which they can rely, depend, or count on, suicidal ideation could emerge (Van Orden et al. 2010).

While somewhat less articulated in the literature, another conceptualization of social integration captures the roles individuals play that make them accountable to others, and from which they derive meaning and purpose. That is, these socially integrated roles (e.g., as marriage partner, parent, caregiver; Stack and Cao 2020) are thought to protect against STBs (Tosti 1898; cf. “reciprocal care” in the Interpersonal Theory [Van Orden et al. 2010]). As such, the loss of one's role—and one's corresponding loss of social integration—could contribute to the development of STBs. For example, the role of a marriage partner traditionally includes care, commitment, service, fidelity, and accountability to one's spouse. When people lose their role of marriage partner via divorce or death of their spouse, they may consequently experience reduced social integration and become at increased risk for STBs (Motillon‐Toudic et al. 2022; Stack 2000). While integration is studied at the group and individual level, within most of the scientific literature and this paper, the term “social integration” refers to readily reaching out to loved ones or community, for example here a spouse. Indeed, in this paper we conceptualize that a person's partner is a part of their community to which they might reach out or connect.

### 1.2 Marital Status and STBs

A recent review paper explored the political, social, cultural, and economic contributions to increasing rates of STBs looking at the roles that people serve, particularly via marital status (Stack 2021). Findings highlighted an association between marital status and STBs. That is, people who were divorced endorsed higher levels of anxiety, depression, substance use, and financial difficulties, compared to people who were married (Stack 2000; Stack and Scourfield 2015). These increased negative outcomes are noteworthy, as each such outcome confers significant risk for STBs (Conwell et al. 2002; Hall et al. 1999; Hempstead and Phillips 2015; Overholser et al. 2011).

Given these findings, it is unsurprising that people who were divorced also exhibited higher STBs than married people (Denney et al. 2015; Stack and Scourfield 2015). Moreover, a meta‐analysis of 170 effect sizes across 36 studies from 2000 to 2016 showed that, on average, the suicide risk for divorced men was four times greater than that for married men, and the suicide risk for divorced women was nearly three times higher than that for married women (Kyung 2018). These data are cross‐sectional and do not imply causal relations, meaning that it is possible that psychopathology symptoms, including anxiety and depression, led to STBs which in turn led marital dissolution, not the other way. However, more recent investigations have supported a longitudinal association between divorce and risk of suicide at follow‐up (e.g., Edwards et al. 2024).

### 1.3 Marital Status and Social Integration

Review papers and individual‐level studies routinely demonstrate a relationship between marital status and social integration (i.e., readily reaching out and connecting with loved ones). Men who were socially well‐integrated had a more than twofold reduced risk of suicide over 24 years follow‐up (Tsai et al. 2015) and women who were socially well‐integrated had a more than threefold lower risk of suicide over 18 years follow‐up (Tsai et al. 2014). Among service members (Braswell and Kushner 2012), community members (Agerbo et al. 2011), and elderly patients (Ertel et al. 2008), married participants as compared to divorced participants endorsed significantly higher levels of social integration. Likewise, across these studies, respondents who reported being divorced endorsed lower levels of social integration. It is important to note however that most investigations into marital status and social integration do not investigate the full spectrum of marital statuses. Instead, research typically compares married versus divorced participants. This leaves considerable question as to the relationship between other marital statuses (e.g., domestic partnership, widowed, single, etc.) with STBs and social integration.

### 1.4 Social Integration and STBs

There is an extensive body of literature demonstrating a link between social integration and STBs. Theories conceptualize social integration as low levels of thwarted belongingness (Van Orden et al. 2010), lack of connection (Klonsky and May 2015), and social problem‐solving deficits (O'Connor and Kirtley 2018); all these constructs involve lessened connections (or perceived connections) with one's community. Indeed, thwarted belongingness, lack of connection, and social problem‐solving deficits are all associated with at least a twofold increase in risk of suicide in a variety of samples, including inpatients, outpatients, military service members, and military Veterans (Chu et al. 2017; Schafer et al. 2021a).

### 1.5 Marital Status, Social Integration, and STBs Among Veterans

The extant literature investigating marital status and STBs does not fully clarify the role of social integration in this relationship. Veteran research provides some insights into this relationship. Increased risk of marital strains is not uncommon in Veterans due to possible residues of the stress of military life coupled with the challenges with transitioning into civilian life post‐service (Wang et al. 2015). Although the literature suggests that rates of divorce do not differ between civilian and active‐duty military personnel (Karney et al. 2012), less is known about divorce in Veteran populations in the years after their service (Routon 2017). Veterans may be more likely to divorce if there are challenges with re‐integration into society, including possible long‐term negative psychological impacts from the time spent in active duty and higher levels of divorce have been seen in Veterans with experience of service deployment versus non‐deployment (Pethrus et al. 2019). It should be noted however that previous research indicates that relationship disruptions (e.g., emotional intimacy, closeness, sexual intimacy, and romance) that occur following deployment are usually transient in nature and typically resolve over a period of months (Knobloch‐Fedders et al. 2020).

Social integration has been identified as a protective factor against STBs among Veteran populations (Schafer et al. 2023). Moreover, compared to Veterans who are divorced or not married, married Veterans have reported greater mental health outcomes, with marriage a protective factor against suicide, exposure to suicide (e.g., of colleagues), and loneliness (Jakupcak et al. 2010; Na et al. 2022; Weisenhorn et al. 2017; Elbogen et al. 2020). In a study of precipitating factors of suicide among Veterans (Kaplan et al. 2012), relationship disruptions were reported among one in every two suicides among Veterans aged 18–34 years. While disruptions in romantic relationships are not always seen in the context of divorce or loss of a spouse to death, this work highlights that disruption and perhaps even the threat of change in relationship status might confer risk for STBs. However, research linking together marital status, social integration, and STBs is scant.

### 1.6 Present Project

The present work explores the cross‐sectional relationships between marital status, social integration, and STBs in Veterans. Theories and extant literature consistently indicate that poor social integration is positively correlated with STBs. Likewise, some literature demonstrates that marital status is also related to STBs. In the present project we investigate the unique cross‐sectional associations of social integration, marital status, and STBs. Understanding the links among these constructs in a Veteran sample in particular is valuable as (1) Veterans experience higher rates of STBs than members of the general population and (2) there are unique factors pertinent to Veteran culture that could intensify the strength of the relationship among marital status, social integration, and STBs in this group. Thus, we used data from the Military Health and Well‐Being Project (Desmarais and Cacace 2022) to investigate four aims among a large sample of military Veterans. (1) We sought to determine if marital status was cross‐sectionally associated with social integration. Consistent with the role‐level approach of social integration, we hypothesized that Veterans who were married as compared to Veterans in all other marital statuses would experience the highest rates of social integration. (2) We investigated if social integration was cross‐sectionally related to STBs in this sample. Consistent with a large body of previous literature and robust findings within that literature (e.g., Stack and Cao 2020), we hypothesized that social integration would be negatively associated with STBs. (3) We determined if marital status was cross‐sectionally related to STBs in this sample. Consistent with previous work, we hypothesized that married Veterans as compared to Veterans with all other marital statuses would experience the highest levels of social integration and that divorced Veterans would experience the lowest levels of social integration. (4) Finally, we investigated if social integration moderated the cross‐sectional link between marital status and STBs. The literature has long assumed that marital status impacts social integration which in turn impacts STBs, but to our knowledge this assumption has not been directly tested in general—much less among a sample of Veterans. In accordance with these past assumptions, we hypothesized that social integration would moderate the cross‐sectional relationship between marital status and STBs.

The present project stands to clarify a timely and interdisciplinary issue in the understanding correlates of STBs. Interdisciplinary investigation into the public health crisis of suicide holds the potential to bring novel solutions to the seemingly relentless and continually elevated suicide rates in the US. Additionally, findings could inform clinical and policy guidance. If social integration is found to moderate the cross‐sectional link between marital status and STBs, then more targeted intervention and prevention efforts to reduce STBs in Veterans with co‐occurring divorce, social integration deficits, and STBs can be developed.

## 2 Methods

### 2.1 Study Design and Procedure

The complete study design and procedures for the Military Health and Well‐Being Project (Desmarais and Cacace 2022) are documented on the Inter‐university Consortium for Political and Social Research website. Further, the data that support the findings of this study are openly available at https://www.icpsr.umich.edu/web/ICPSR/studies/38304. Researchers used Qualtrics Panels to collect a stratified sample of United States Military Veterans who served in post‐Vietnam war eras. Participants were recruited through multiple websites. Then, again using Qualtrics Panel, participants were screened to ensure that they met the inclusion criteria which were military service, included age minimum (*min* = 18 years), service era (i.e., post‐Vietnam era), and U.S. residence. To enhance representation of minoritized identities in military Veterans, Black military Veterans and female Veterans were oversampled.

Individuals who met all inclusion criteria were eligible to complete the survey. A total of 1,863 responses were collected. In total, 1,522 participants answered all questions, met the inclusion requirements (i.e., were Veterans who served during the post‐Vietnam era), and completed the survey within three standard deviations of the average time to complete. 27 participants were excluded for not reporting their age. A further 26 participants were excluded for not fully completing the key measures of interest (i.e., measures of marital status, social integration, or STBs).

The present sample has been used to explore many associations and phenomena. For example, the authors of this paper have investigated links between social integration, traumatic brain injuries, and STBs (Schafer et al. 2023), loneliness and quality of life (Schafer et al. 2023), risk and protective factors of STBs (Schafer et al. 2024), loneliness, substance use, and STBs (Schafer et al. 2024), race and gender with STBs (Schafer et al. under review), and loneliness as a mediator between combat trauma and STBs (Schafer et al. 2024). Those manuscripts provide insight into unique aspects that may be associated with increased incidences of STBs in Veterans, but they do not directly speak to the burden of STBs between Veterans of varying marital status, nor do they address the specific role of moderation studied here. The role of the present manuscript is to determine if STBs and social integration may vary based on marital status in Veterans, expand the literature on this topic from where it presently is— comparing almost exclusively married versus single Veterans. Further, we seek to examine the moderating role of social integration in link between the marital status and STBs. Previous work using this dataset instead largely centered on the relation between traumatic brain injuries, substance use, and loneliness with STBs. Further, previous work combined all four STB outcomes into a single measure of “suicidality” while this paper adds the unique contribution of more nuanced investigation into each STB outcome: lifetime suicidal ideation, past year suicidal ideation, communication of suicidal ideation, and likelihood of future suicide attempts.

### 2.2 Participants

U.S. Military Veterans were recruited between May 2020 through June 2020. The goal of the overall project was to collect information regarding psychosocial correlates of health and wellness, including military identity, self‐stigma, daily stress, combat exposure, purpose and value, substance use, traumatic brain injury, moral injury, suicide risk, social integration and contribution, and the Substance Abuse and Mental Health Services Administration dimensions of wellness (i.e., social, emotional, spiritual, intellectual, physical, and environmental components for this study).

### 2.3 Measures

#### 2.3.1 Marital Status

Participants were asked to select the answer that best described their marital status at the time of the survey. There were five items from which to choose (1 = *Single*, 2 = *Married*, 3 = *Domestic partnership*, 4 = *Divorced*, 5 = *Widowed*).

#### 2.3.2 Social Well‐Being: Social Integration Subscale (Keyes 1998)

Social integration was measured on a Likert scale from 1 (*Strongly disagree*) to 7 (*Strongly agree*) using the following three questions: “I don't feel I belong to anything I'd call a community” (reverse‐coded), “I feel close to other people in my community,” and “My community is a source of comfort.” A mean score was computed, with higher scores indicating greater social integration. Within this sample, Cronbach's α was 0.79, indicating adequate internal consistency. Previous research indicates that gender is significantly related to social integration (Dalgard and Thapa 2007).

#### 2.3.3 Suicidal Behavior Questionnaire‐Revised (SBQ‐R; Osman et al. 2001)

The SBQ‐R is a four‐item self‐report questionnaire that measures four dimensions of suicidality: lifetime suicidal ideation, past year suicidal ideation, threat of suicide attempt, and self‐assessed likelihood of future suicidal behavior. The first item was, “Have you ever thought about or attempted to kill yourself?”, with answers scored on a six‐point Likert scale from 1 (*Never*) to 6 (*I have attempted to kill myself, and I really hoped to die*). The second item was, “How often have you thought about killing yourself in the past year?”, with responses ranging from 1 (*Never*) to 5 (*Very Often*). The third item was, “Have you ever told someone that you were going to [die by] suicide or that you might try to kill yourself?”, with possible responses rated from 1 (*No*) to 5 (*Yes, more than once, and really wanted to do it*). The fourth question was, “How likely is it that you will attempt suicide someday?”, with responses ranging from 0 (*Never*) to 6 (*Very likely*). A total score was calculated across the four items, with higher scores indicating more intense suicidality. The SBQ‐R has been validated previously within military populations (Franks et al. 2021). Within this sample, Cronbach's α was 0.89, reflecting good internal consistency.

### 2.4 Data Analytic Plan

All statistics were conducted in SPSS 27 (IBM 2023). To begin we conducted *descriptive statistics* on study‐relevant variables: gender, age, race, marital status, social integration, and the four STB outcomes. Then, we constructed a Pearson *correlation* matrix to study the cross‐sectional associations between age, gender, social integration, and the four STB outcomes: lifetime suicidal ideation, past year suicidal ideation, communication of suicidal ideation, and likelihood of future suicide attempts. STB outcomes are coded with responses ranging from 0 (Never) to 6 (Very likely). For all ANOVAs, marital status is coded as 1 = *Single*, 2 = *Married*, 3 = *Domestic partnership*, 4 = *Divorced*, 5 = *Widowed*. All data are cross‐sectional.

- Our *first aim* was to test for differences in social integration between marital status using a one‐way ANOVA with post‐hoc contrast via Tukey's test. We sought to determine if social integration varied based on marital status.
- Our *second aim* was to investigate the cross‐sectional associations between social integration and lifetime suicidal ideation, past year suicidal ideation, communication of suicidal ideation, and likelihood of future suicide attempt using a correlational approach. This was conducted by examining the Pearson correlation matrix.
- Our *third aim* was to determine if lifetime suicidal ideation, past year suicidal ideation, communication of suicidal ideation, and likelihood of future suicide attempt varied by marital status, using a series of one‐way ANOVAs with post‐hoc contrast via Tukey's tests.
- Our *fourth aim* was to determine if social integration moderated the cross‐sectional link between marital status and STB outcomes, using the PROCESS Macro. We used four models, one for each STB outcome. PROCESS Macros was set to model 1 (i.e., simple moderation with a continuous variable as the moderator) with 5,000‐unit bootstrapping. Results are reported at a 95% confidence interval. Marital status was the independent variable. Mean social integration score was the moderating variable.

## 3 Results

### 3.1 Descriptive Statistics

Descriptive statistics are depicted in Table 1. They demonstrate that the sample was diverse regarding marital status, gender, and race. Indeed, regarding gender, this sample reflects elevated representation of female Veterans as respective to their prevalence in the US Veteran population. That is, while female Veterans account for a little more than 10% of the US Veteran population, a third of this sample is female (VA.gov). Similarly, this sample strongly reflects representation of Black and African American Veterans who comprise 12% of the US Veteran population, yet here account for 14% of the sample (VA.gov).

**TABLE 1:** Descriptive statistics.

		*n*	*%*		
Gender	Male	985	67.1		
	Female	476	32.4		
	Transgender/non‐binary/prefer not to say	8	0.5		
	Total	1469	100		
Race	White	1109	75.5		
	Black or African American	211	14.4		
	Hispanic or Latino	84	5.7		
	Asian	42	2.9		
	American Indian or Alaska Native	13	0.9		
	Native Hawaiian or other Pacific Islander	10	0.7		
	Total	1469	100		
Marital Status	Single	279	19.0		
	Married	864	58.8		
	Domestic partnership	73	5.0		
	Divorced	210	14.3		
	Widowed	43	2.9		
	Total	1,469	100		

		*min*	*max*	*M*	SD
	Age	18	86	50.08	13.37
	Social integration	1	6	3.772	0.94
	Lifetime suicidal ideation	1	6	1.75	1.17
	Past year suicidal ideation	1	5	1.51	0.97
	Suicidal ideation communication	1	5	1.35	0.85
	Likelihood of future suicide attempt	0	6	0.69	1.23

Table 2 depicts social integration and STB outcomes by marital status. These statistics indicate that STB outcomes are present across the entire sample. SBQ‐R total scores of eight and above are generally considered “suicidal samples” (Osman et al. 2001), and our sample's median SBQ‐R score was just above five. This indicates that the average participant endorsed at least some lifetime suicidal ideation, past year suicidal ideation, communication of suicidal ideation, and likelihood of future suicide attempt. Likewise, responses related to the three‐item social integration measure demonstrated that within this sample Veterans endorsed relatively low levels of social integration. Scores related to social integration items vary from 3 to 21, with lower scores reflecting lower social integration. Within this sample, the mean total score was 3.77 (SD = 0.94, median = 4.00), indicating that on the whole these Veterans frequently endorsed little to no social integration on these items. There are no suggested cutoffs for this measure, thus it is not possible to articulate cutoffs between samples that are or are not socially integrated. Nevertheless, a sample endorsing a mean social integration score at the low end of the measure likely reflects significant deficits in this domain.

**TABLE 2:** Means and standard deviations of social integration and suicidal thoughts and behaviors by marital status.

Marital Status		Social integration		Suicidal thoughts and behaviors		Lifetime suicidal ideation		Past year suicidal ideation		Communication of suicidal ideation		Likelihood of future suicide attempt	
	*N*	*M*	SD	*M*	SD	*M*	SD	*M*	SD	*M*	SD	*M*	SD
Single	279	3.77	0.97	6.11	4.3	1.90	1.27	1.71	1.11	1.52	1.03	0.97	1.44
Married	864	3.83	0.91	4.84	3.26	1.62	1.05	1.4	0.85	1.27	0.73	0.55	1.12
Domestic partnership	73	3.76	1.06	7.3	4.69	2.30	1.51	2.05	1.31	1.74	1.21	1.21	1.49
Divorced	210	3.54	0.94	5.43	3.86	1.88	1.32	1.51	1.03	1.34	0.9	0.7	1.23
Widowed	43	3.62	0.96	5.23	3.35	1.72	1.12	1.49	0.86	1.28	0.73	0.74	1.09
Total	1,469	3.77	0.94	5.3	3.7	1.75	1.18	1.51	0.97	1.35	0.86	0.69	1.24

*Note*: Suicidal thoughts and behaviors = SQB‐R total score; Lifetime suicidal ideation = SBQ‐R item 1; Past year suicidal ideation = SBQ‐R item 2; Communication of suicidal ideation = SBQ‐R item 3; Likelihood of future suicide attempt = SBQ‐R item 4.

Abbreviations: *M* = mean score, SD = standard deviation.

### 3.2 Correlations

Pearson bivariate correlations are displayed in Table 3. This table indicates that all four STB outcomes were significantly and positively correlated with gender, meaning that all four STB outcomes were more likely to be endorsed by female Veterans. Contrasting this, STB outcomes were significantly and *negatively* associated with age and social integration, such that older Veterans were much less likely to endorse STB outcomes. Likewise, social integration was significantly negatively correlated with age and every STB outcome, meaning that Veterans who endorsed higher social integration endorsed fewer STBs. Further, older Veterans reported significantly lower social integration.

**TABLE 3:** Correlation matrix.

	1	2	3	4	5	6	7
1 Gender	1						
2 Age	−0.16*	1					
3 Social integration	0.01	−0.096*	1				
4 Lifetime suicidal ideation	0.18*	−0.20*	−.019*	1			
5 Past year suicidal ideation	0.15*	−0.25*	−0.16*	0.75*	1		
6 Communication of suicidal ideation	0.15*	−0.23*	−0.09*	0.67*	0.62*	1	
7 Likelihood of future suicide attempt	0.15*	−0.22*	−0.12*	0.69*	0.74*	0.60*	1

* Correlation is significant at the 0.01 level (2‐tailed). Gender is coded as 1 = Male, 2 = Female, 3 = transgender, non‐binary, prefer not to say.

### 3.3 Social Integration by Marital Status

Our *first aim* was to determine if social integration varied by marital status. We did this using a one‐way ANOVA with post‐hoc contrast via Tukey's test. The model was significant (*F* [4,1] = 4.15, *p* = 0.002) indicating that there were significant differences in social integration based on marital status. Analysis of the Tukey's post hoc comparisons table indicated that the only differences in social integration by marital status were between married and divorced participants such that married participants (*M* = 3.83) reported significantly higher mean social integration scores than divorced participant (*M* = 3.54, *p* < 0.001).

### 3.4 Social Integration and STBs

Our *second aim* was to investigate the association between social integration and lifetime suicidal ideation, past year suicidal ideation, communication of suicidal ideation, and likelihood of future suicide attempt using a correlational approach. Results from the correlation matrix indicated that social integration was negatively and significantly correlated with all four STB outcomes: lifetime suicidal ideation (*r* = −0.19, *p* < 0.001), past year suicidal ideation (*r* = −0.16, *p* < 0.001), communication of suicidal ideation (*r* = −0.09, *p* < 0.001), and likelihood of future suicide attempt (*r* = 0.12, *p* < 0.001). This indicated higher social integration scores were correlated with lower STB scores.

### 3.5 STBs by Marital Status

Our *third* aim was to determine if lifetime suicidal ideation, past year suicidal ideation, communication of suicidal ideation, and likelihood of future suicide attempt varied based on marital status, using a series of one‐way ANOVAs with each STB outcome as a dependent variable. All one‐way ANOVAs included post‐hoc contrast via Tukey's tests.

#### 3.5.1 Lifetime Suicidal Ideation

The first item of the SBQ‐R reads: Have you ever thought about or attempted to kill yourself? Findings indicated that there were significant differences in lifetime suicidal ideation by marital status (*F* [4,1] = 8.68, *p* < 0.001). People who were single (*M* = 1.90) and people who were in a domestic partnership (*M* = 2.30) reported significantly higher levels of lifetime suicidal ideation than people who were married (*M* = 1.62) with a statistical significance of *p* < 0.001.

#### 3.5.2 Past Year Suicidal Ideation

The second item of the SBQ‐R reads: How often have you thought about killing yourself in the past year? We investigated if this item measuring past year suicidal ideation varied by marital status. This one‐way ANOVA indicated that there were significant differences in levels of past year suicidal ideation by marital status (*F* [4,1] = 11.96, *p* < 0.001) such that people who were in a domestic partnership (*M* = 2.05) reported higher frequency of past year suicidal ideation than people who were married (*M* = 1.40), divorced (*M* = 1.51), or widowed (*M* = 1.49) at a *p* < 0.001 level.

#### 3.5.3 Suicidal Ideation Communication

The third item of the SBQ‐R is: Have you ever told someone that you were going to commit suicide or that you might do it? We investigated if this item—that is, suicidal ideation communication—varied by marital status. Findings from this one‐way ANOVA indicated again significant differences in the communication of suicidal ideation (*F* [4,1] = 8.80, *p* < 0.001). People who were in a domestic partnership (*M* = 1.74) reported that they were significantly more likely to have communicated suicidal ideation to other people as compared to people who were married (*M* = 1.27), divorced (*M* = 1.34), or widowed (*M* = 1.28) at a *p* < 0.001 level.

#### 3.5.4 Likelihood of Future Suicide Attempt

The final item of the SBQ‐R is: How likely is it that you would attempt suicide some day? We investigated if this item—the likelihood of future suicide attempt—varied by marital status. Results from this one‐way ANOVA indicated that there were significant differences in the likelihood of future suicide attempt based on marital status (*F* [4,1] = 9.58, *p* < 0.001). Post hoc comparisons indicated that people who were in a domestic partnership (*M* = 1.21) were significantly more likely to attempt suicide in the future as compared to people who were married (*M* = 0.55) or divorced (*M* = 0.70). Likewise, people who were single (*M* = 0.97) were also more likely to attempt suicide in the future as compared to people who were married (*M* = 0.55). All differences are significant at the *p* < 0.001 level.

### 3.6 Social Integration as a Moderator Between Marital Status and STBs

Our fourth and final aim was to investigate social integration as a moderator in the cross‐sectional relation between marital status and each of the four STB outcomes. Indeed, previous literature largely assumes that marital status impacts social integration which in turn impacts STBs. Thus, this assumption is the inspiration for the present analyses, which approximates these relationships in a cross‐sectional manner. We employed four PROCESS Macro models (simple moderation, model 1) controlling for race, age, gender, and income. Results are listed below.

#### 3.6.1 Lifetime Suicidal Ideation

**FIGURE 1:**
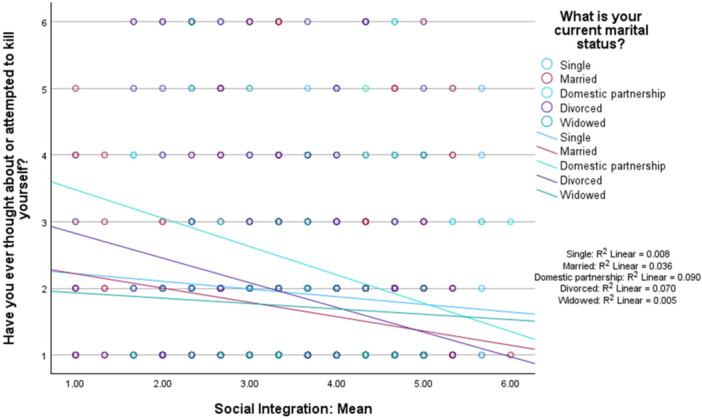
Social integration as a moderator between marital status and lifetime suicidal ideation.

Social integration was a not significant moderator in the link between marital status and lifetime suicidal ideation (omnibus test: *R* ^2^ = 0.112, *MSE* = 1.22, *F* [3,1465] = 26.65, interaction effects: *p* < 0.001, *B* = ‐0.047, *p* = 0.11). This is depicted in Figure 1.

#### 3.6.2 Past Year Suicidal Ideation

**FIGURE 2:**
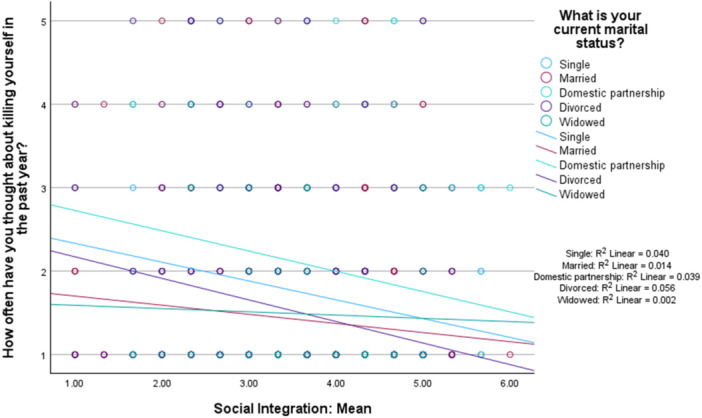
Social integration as a moderator between marital status and past year suicidal ideation.

Likewise, social integration was a not significant moderator between marital status and past year suicidal ideation (omnibus test: *R* ^*2*^ = 0.027, *MSE* = 0.92, *F* [3,1465] = 13.45, *p* < 0.001, interaction effects: *B* = 0.01, *p* = 0.84). These findings are shown in Figure 2.

#### 3.6.3 Suicidal Ideation Communication

**FIGURE 3:**
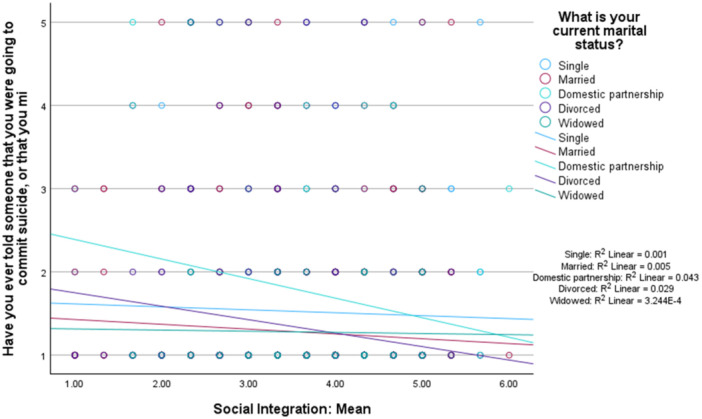
Social integration as a moderator between marital status and suicidal ideation communication.

Social integration was not a significant moderator in the association between marital status and communication of suicidal ideation (omnibus test: *R* ^*2*^ = 0.01, *MSE* = 0.73, *F* [3,1465] = 5.18, *p* = 0.014, interaction effects: *B* = ‐0.004, *p* = 0.94), and the related figure is shown in Figure 3.

#### 3.6.4 Likelihood of Future Suicide Attempt

**FIGURE 4:**
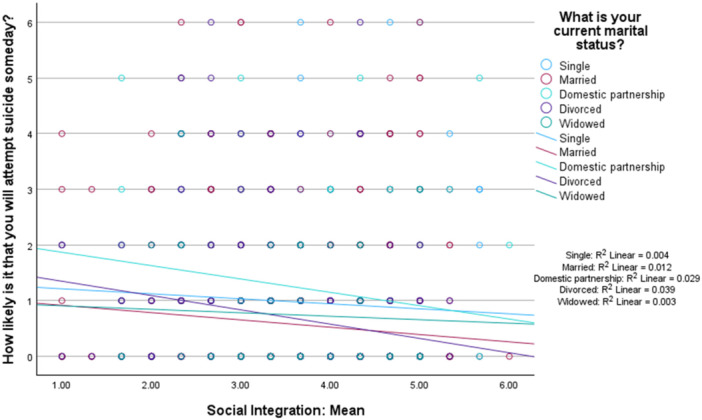
Social integration as a moderator between marital status and likelihood of suicide attempt.

Social integration was not significant a moderator in the link between marital status and the likelihood of future suicide attempt significant moderator (omnibus test: *R* ^*2*^ = 0.015, *MSE* = 1.52, *F* [3,1465] = 7.45, *p* = 0.001, interaction effects: *B* = −0.062, *p* = 0.46). Findings are shown in Figure 4.

## 4 Discussion

In the present study we investigated the cross‐sectional link between marital status, social integration, and STBs among a sample of military Veterans. STBs were conceptualized as lifetime suicidal ideation, past year suicidal ideation, communication of suicidal ideation, and the likelihood of future suicide attempt. Our study was comprised of four key aims. We sought to (1) test for differences in social integration based on marital status, (2) investigate the associations between social integration and lifetime suicidal ideation, past year suicidal ideation, communication of suicidal ideation, and likelihood of future suicide attempt, (3) determine if lifetime suicidal ideation, past year suicidal ideation, communication of suicidal ideation, and likelihood of future suicide attempt varied by marital status, and (4) determine if social integration moderated the cross‐sectional link between marital status and STB outcomes.

Within our study, (1) marital status was associated with social integration such that married participants had higher social integration than did single participants. (2) Social integration was likewise related to STBs: as social integration decreased, the presence of lifetime suicidal ideation, past year suicidal ideation, communication of suicidal ideation, and the likelihood of future suicide attempts all increased. (3) STBs varied by marital status such that single participants were more likely than married participants to endorse suicidal ideation; participants who were in a domestic partnership were more likely to have past year suicidal ideation and have communicated suicidal ideation to other people as compared to participants who were married, divorced, or widowed; participants in a domestic partnership were also significantly more likely to attempt suicide in the future as compared to people who were married or divorced. Likewise, people who were single were also more likely to attempt suicide in the future as compared to people who were married. Finally, (4) social integration did not moderate the link between marital status and any of the STB outcomes.

These findings were somewhat consistent with our hypotheses, and they show that STBs vary based on social integration which in turn varies based on marital status. However, given that social integration did not act as a moderator in the link between marital status and STBs, the field should consider that factors external to the link between marital status and social integration contribute to the variance in STBs. Indeed, these findings might show that as the field of psychology seeks to understand what might contribute to the development of STBs, marital status on its own might be a less specific approach. Instead, the level of social integration (e.g., the degree to which a respondent is closely connected to their romantic partner) may account for much of the variance of STBs.

### 4.1 Theory Implications

Researchers have long proposed that psychiatric, psychological, and biological factors are associated with suicide. There is an historical tradition of examining the social determinants of suicide, led by Durkheim, who proposed that STBs is inversely correlated with social integration (see, e.g., Tosti 1898). Psychological theories (Klonsky and May 2015; O'Connor and Kirtley 2018; Van Orden et al. 2010) proposed that poor social integration—conceptualized as thwarted belongingness, lack of connection, or low levels of social connection as well as the lack of people relying on us in the roles that we play—leads to STBs. Work from decades of literature support these suppositions and show that married people have higher rates of social integration (Barton et al. 2014; Stevens and Westerhof 2006; Trudel‐Fitzgerald et al. 2019), which in turn are linked with lower rates of STBs (Handley et al. 2012; Stack and Cao 2020; Tsai et al. 2015). While previous work demonstrated a robust relation between marital status, social integration, and STBs, the moderating role of social integration was unclear. Our findings are consistent with theories suggesting that STBs and social integration vary based on marital status. Likewise, there is support for aspects of theories (Klonsky and May 2015; O'Connor and Kirtley 2018; Van Orden et al. 2010) which hypothesize that thwarted belongingness, and difficulty in being able to rely on others, are associated with STBs. The key takeaway from our work is that social integration, rather than marital status itself, seems to be of primary importance in STBs.

### 4.2 Clinical Implications

This work is merely observational, with no intervention or treatment provided. As such, clinical implications are softened, but nevertheless this work suggests that social integration could be a viable treatment target for Veterans who present with STBs within a variety of marital statuses. In other words, based on the present work, STBs may have less to do with marital status alone, and more to do with how well (or poorly) Veterans feel integrated with others in their community, including their romantic partner. This is particularly relevant for Veterans, a group that has important social integration facets. For example, military‐related samples have been studied for unit cohesion (Gallyer et al. 2020), social closeness (Dempsey et al. 2021), and social responsibility (Nasih et al. 2019). Similarly, while engaged with military service, Veterans were relied on by loved ones for income, health insurance, and security. These aspects of social integration, whether measured as reliance on or from the service member, are heightened in or unique to military samples (Routon 2017). Compounding that, upon separating from the military some Veterans may experience rapid decline in social integration again whether conceptualized from the sociological or psychological framework (Routon 2017). We recommend that clinicians target and bolster social integration as this may restore Veterans with new onset STBs to their prior level of functioning. Working to integrate Veterans into their environment could reduce or prevent the development of STBs.

### 4.3 Policy Implications

Veterans exhibit elevated rates of STBs, and there is public interest on the national level to engage in policy efforts to reduce this STBs. The findings from the present project suggest that having a space for Veterans to feel included and supported (whether or not they are experiencing marital distress) may help reduce or prevent STBs in Veterans. From a policy perspective, there should be efforts to transition people from active service into a fully socially integrated Veteran life. The military spends substantial time and money integrating new Service members into military life. Service members spend weeks in basic training, complete coursework, and are entrenched in military values and culture. However, the military currently plays a lesser role in helping its service members assimilate back into civilian life as Veterans. The work we present here suggests that integrating and assimilating Veterans into an active, welcoming, purpose‐filled community could reduce the risk of STBs among this population.

### 4.4 Limitations

While the present study advances our knowledge in this area as it features a diverse sample measured across a variety of relevant variables, our findings should be interpreted in light of some limitations. For example, these data are cross‐sectional, which reduces our ability to draw inferences on the development of STBs over time and precludes us from making temporal and causal claims about the associations among marital status, social integration, and STBs. Although we assume, on theoretical grounds, that the directionality of the associations we detected goes from marital status to social integration to STBs, other explanations are possible, and the inclusion of additional variables in the model may alter findings. It is possible, for instance, that marital status is affected by social integration and STBs. Thus, although we assume directionality and temporal antecedence, the available data do not allow us to perform a strong test of these assumptions. Additionally, the measure of social integration is somewhat vague in that it does not specify a timepoint, making it difficult to ascertain whether Veterans' social integration scores indicate acute or longstanding social integration (or, conversely, an acute or chronic lack of social integration). There may be variability in the way that participants interpreted the social integration questions.

There are also issues with generalizability. First, these data were collected from an exclusive sample of Veterans. Thus, it is possible that the unique stressors and cultural norms within military personnel and Veterans precludes generalization of findings to a broader population. Likewise, these data were collected during the early months of the COVID‐19 pandemic and related lockdowns, which may have presented a more intense experience of marital strain, social integration deficits, and STBs (see Schafer et al. 2020; 2021b, for a review of elevated rates of psychopathology during the pandemic as compared to immediately prior to the pandemic).

### 4.5 Conclusions

We studied the relationship between marital status, social integration (i.e., readily reaching out to loved ones and other support networks), and STBs. The specific interrelations among marital status, social integration, and STBs among Veterans have not been studied before to our knowledge; thus, we sought to make a preliminary test of these interrelations and, in particular, of the potential moderating role of social integration between marital status and STBs, in accordance with longstanding sociological and psychological theories. Findings indicated that marital status was associated with social integration. Veterans who were married displayed significantly higher rates of social integration as compared to all other Veterans. Social integration in turn was associated with STBs such that higher levels of social integration were associated with lower levels of STBs. STBs were found to vary by marital status, such that married Veterans reported lower STBs than Veterans who were single. These findings suggest that variance in STBs may be accounted for more so based on social integration and less so based on marital status. Based on the data from the Military Health and Well‐Being Project, we recommend that clinicians who treat Veterans presenting with co‐occurring social integration difficulties and STBs treat the social integration, regardless of marital status. Intentionally supporting Veterans to become socially integrated within their communities, friend‐groups and families, could effectively protect against STBs and improve outcomes.

## Ethics Statement

Given that all data came to the authors de‐identified, this study has been found to not contain human subjects and has been found to be exempt from IRB approval.

## Data Availability

These data are freely available at the University of Michigan ICPSR website https://www.icpsr.umich.edu/web/ICPSR/studies/38304/summary. The data that support the findings of this study are openly available in Military Health and Well‐Being Project, United States, 2020 at https://www.icpsr.umich.edu/web/ICPSR/studies/38304/publications, reference number (ICPSR 38304).

## References

[jclp70093-bib-0001] Agerbo, E. , S. Stack , and L. Petersen . 2011. “Social Integration and Suicide: Denmark, 1906–2006.” Social Science Journal 48, no. 4: 630–640.

[jclp70093-bib-0002] Barton, A. W. , T. G. Futris , and R. B. Nielsen . 2014. “With a Little Help From Our Friends: Couple Social Integration in Marriage.” Journal of Family Psychology 28, no. 6: 986–991. 25485674 10.1037/fam0000038

[jclp70093-bib-0051] Beck, A. T. , M. Kovacs , and A. Weissman . 1975. “Hopelessness and Suicidal Behavior: An Overview.” JAMA 234, no. 11: 1146–1149. 1242427

[jclp70093-bib-0003] Braswell, H. , and H. I. Kushner . 2012. “Suicide, Social Integration, and Masculinity in the US Military.” Social Science & Medicine (1982) 74, no. 4: 530–536. 21036443 10.1016/j.socscimed.2010.07.031

[jclp70093-bib-0055] Chu, C. , J. M. Buchman‐Schmitt , I. H. Stanley , et al. 2017. “The Interpersonal Theory of Suicide: A Systematic Review and Meta‐Analysis of a Decade of Cross‐National Research.” Psychological Bulletin 143, no. 12: 1313. 29072480 10.1037/bul0000123 PMC5730496

[jclp70093-bib-0004] Conwell, Y. , P. R. Duberstein , and E. D. Caine . 2002. “Risk Factors for Suicide in Later Life.” Biological Psychiatry 52, no. 3: 193–204. 10.1016/s0006-3223(02)01347-1. 12182926

[jclp70093-bib-0056] Dalgard, O. S. , and S. B. Thapa . 2007. “Immigration, Social Integration and Mental Health in Norway, With Focus on Gender Differences.” Clinical Practice and Epidemiology in Mental Health 3, no. 1: 24. 17971211 10.1186/1745-0179-3-24 PMC2222607

[jclp70093-bib-0005] Dempsey, C. L. , D. M. Benedek , M. K. Nock , et al. 2021. “Social Closeness and Support Are Associated With Lower Risk of Suicide Among US Army Soldiers.” Suicide and Life‐Threatening Behavior 51, no. 5: 940–954. 34196966 10.1111/sltb.12778 PMC10615249

[jclp70093-bib-0053] Denney, J. T. , T. Wadsworth , R. G. Rogers , and F. C. Pampel . 2015. “Suicide in the City: Do Characteristics of Place Really Influence Risk?” Social Science Quarterly 96, no. 2: 313–329. 26236047 10.1111/ssqu.12165 PMC4519975

[jclp70093-bib-0006] Desmarais, S. L. , and S. Cacace . Military Health and Well‐Being Project, United States, 2020. Inter‐University Consortium for Political and Social Research [distributor], 2022‐02‐09. 10.3886/ICPSR38304.v1.

[jclp70093-bib-0054] Edwards, A. C. , H. Ohlsson , J. E. Salvatore , et al. 2024. “Divorce and Risk of Suicide Attempt: A Swedish National Study.” Psychological Medicine 54, no. 8: 1620–1628. 38084643 10.1017/S0033291723003513 PMC11551852

[jclp70093-bib-0009] Elbogen, E. B. , K. Molloy , H. R. Wagner , et al. 2020. “Psychosocial Protective Factors and Suicidal Ideation: Results From a National Longitudinal Study of Veterans.” Journal of Affective Disorders 260: 703–709. 31561113 10.1016/j.jad.2019.09.062 PMC6878990

[jclp70093-bib-0010] Ertel, K. A. , M. M. Glymour , and L. F. Berkman . 2008. “Effects of Social Integration on Pre Serving Memory Function in a Nationally Representative US Elderly Population.” American Journal of Public Health 98, no. 7: 1215–1220. 18511736 10.2105/AJPH.2007.113654 PMC2424091

[jclp70093-bib-0057] Franks, M. , R. J. Cramer , C. A. Cunningham , A. R. Kaniuka , and C. J. Bryan . 2021. “Psychometric Assessment of Two Suicide Screeners When Used Under Routine Conditions in Military Outpatient Treatment Programs.” Psychological Services 18, no. 3: 433. 32118461 10.1037/ser0000416

[jclp70093-bib-0059] Gallyer, A. J. , I. H. Stanley , T. N. Day , and T. E. Joiner . 2020. “Examining the Interaction of Autism Spectrum Disorder‐Related Traits and Unit Cohesion on Suicide Risk Among Military Personnel.” Journal of Affective Disorders 271: 59–65. 32312698 10.1016/j.jad.2020.03.092 PMC7812611

[jclp70093-bib-0011] Hall, R. C. W. , D. E. Platt , and R. C. W. Hall . 1999. “Suicide Risk Assessment: A Review of Risk Factors for Suicide in 100 Patients Who Made Severe Suicide Attempts.” Psychosomatics 40, no. 1: 18–27. 10.1016/s0033-3182(99)71267-3. 9989117

[jclp70093-bib-0012] Handley, T. E. , K. J. Inder , B. J. Kelly , et al. 2012. “You've Got to Have Friends: The Predictive Value of Social Integration and Support in Suicidal Ideation Among Rural Communities.” Social Psychiatry and Psychiatric Epidemiology 47: 1281–1290. 21989656 10.1007/s00127-011-0436-y

[jclp70093-bib-0013] Hempstead, K. A. , and J. A. Phillips . 2015. “Rising Suicide Among Adults Aged 40–64 Years.” American Journal of Preventive Medicine 48, no. 5: 491–500. 10.1016/j.amepre.2014.11.006. 25736978

[jclp70093-bib-0058] IBM Corp . 2023. IBM SPSS Statistics for Windows (Version 27.0) [Computer software]. IBM Corp.

[jclp70093-bib-0014] Jakupcak, M. , S. Vannoy , Z. Imel , et al. 2010. “Does PTSD Moderate the Relationship Between Social Support and Suicide Risk in Iraq and Afghanistan War Veterans Seeking Mental Health Treatment?” Depression and Anxiety 27, no. 11: 1001–1005. 20721901 10.1002/da.20722 PMC3038554

[jclp70093-bib-0015] Kaplan, M. S. , B. H. McFarland , N. Huguet , and M. Valenstein . 2012. “Suicide Risk and Precip Itating Circumstances Among Young, Middle‐Aged, and Older Male Veterans.” American Journal of Public Health 102, no. S1: S131–S137. 22390587 10.2105/AJPH.2011.300445 PMC3496453

[jclp70093-bib-0016] Karney, B. R. , D. S. Loughran , and M. S. Pollard . 2012. “Comparing Marital Status and Divorce Status in Civilian and Military Populations.” Journal of Family Issues 33, no. 12: 1572–1594.

[jclp70093-bib-0017] Keyes, C. L. M. 1998. “Social Well‐Being.” Social Psychology Quarterly 61: 121–140.

[jclp70093-bib-0018] Klonsky, E. D. , and A. M. May . 2015. “The Three‐Step Theory (3ST): A New Theory of Suicide Rooted in the “Ideation‐To‐Action” Framework.” International Journal of Cognitive Therapy 8, no. 2: 114–129.

[jclp70093-bib-0019] Knobloch‐Fedders, L. M. , L. K. Knobloch , S. Scott , and H. Fiore . 2020. “Relationship Changes of Military Couples During Reintegration: A Longitudinal Analysis.” Journal of Social and Personal Relationships 37, no. 7: 2145–2165.

[jclp70093-bib-0020] Kyung‐Sook, W. , S. SangSoo , S. Sangjin , and S. Young‐Jeon . 2018. “Marital Status Integration and Suicide: A Meta Analysis and Meta Regression.” Social Science & Medicine 197: 116–126. 29227910 10.1016/j.socscimed.2017.11.053

[jclp70093-bib-0021] Motillon‐Toudic, C. , M. Walter , M. Séguin , J. D. Carrier , S. Berrouiguet , and C. Lemey . 2022. “Social Isolation and Suicide Risk: Literature Review and Perspectives.” European Psychiatry 65, no. 1: e65. 36216777 10.1192/j.eurpsy.2022.2320 PMC9641655

[jclp70093-bib-0022] Na, P. J. , E. Straus , T. Jack Tsai , S. B. Norman , S. M. Southwick , and R. H. Pietrzak . 2022. “Loneliness in US Military Veterans During the COVID‐19 Pandemic: A Nationally Representative, Prospective Cohort Study.” Journal of Psychiatric Research 151: 546–553. 35636030 10.1016/j.jpsychires.2022.05.042 PMC9126310

[jclp70093-bib-0023] Nasih, M. , I. Harymawan , F. K. G. Putra , and R. Qotrunnada . 2019. “Military Experienced Board and Corporate Social Responsibility Disclosure: An Empirical Evidence From Indonesia.” Entrepreneurship and Sustainability Issues 7, no. 1: 553–573.

[jclp70093-bib-0024] O'Connor, R. C. , and O. J. Kirtley . 2018. “The Integrated Motivational–Volitional Model of Suicidal Behaviour.” Philosophical Transactions of the Royal Society B: Biological Sciences 373, no. 1754: 20170268. 10.1098/rstb.2017.0268 PMC6053985 30012735

[jclp70093-bib-0025] Van Orden, K. A. , T. K. Witte , K. C. Cukrowicz , S. R. Braithwaite , E. A. Selby , and T. E. Joiner, Jr. . 2010. “The Interpersonal Theory of Suicide.” Psychological Review 117, no. 2: 575–600. 20438238 10.1037/a0018697 PMC3130348

[jclp70093-bib-0026] Osman, A. , C. L. Bagge , P. M. Gutierrez , L. C. Konick , B. A. Kopper , and F. X. Barrios . 2001. “The Suicidal Behaviors Questionnaire‐Revised (SBQ‐R): Validation With Clinical and Nonclinical Samples.” Assessment 8, no. 4: 443–454. 11785588 10.1177/107319110100800409

[jclp70093-bib-0027] Overholser, J. C. , A. Braden , and L. Dieter . 2011. “Understanding Suicide Risk: Identification of High‐Risk Groups During High‐Risk Times.” Journal of Clinical Psychology 68, no. 3: 349–361. 10.1002/jclp.20859. 22140004 PMC3379545

[jclp70093-bib-0028] Pethrus, C. M. , J. Reutfors , K. Johansson , et al. 2019. “Marriage and Divorce After Military Deployment to Afghanistan: A Matched Cohort Study From Sweden.” PLoS One 14, no. 2: e0207981. 30707702 10.1371/journal.pone.0207981 PMC6358058

[jclp70093-bib-0030] Routon, P. W. 2017. “Military Service and Marital Dissolution: A Trajectory Analysis.” Review of Economics of the Household 15: 335–355.

[jclp70093-bib-0049] Schafer, K. , G. A. Kennedy , and T. E. Joiner . 2020. "Hopelessness, Interpersonal, and Emotion Regulation Perspectives on Suicidal Ideation: Tests in a High Risk Clinical Sample." Archives of Suicide Research. 10.1080/13811118.2020.1859031 33336628

[jclp70093-bib-0032] Schafer, K. M. , G. Kennedy , A. Gallyer , and P. Resnik . 2021a. “A Direct Comparison of Theory‐Driven and Machine Learning Prediction of Suicide: A Meta‐Analysis.” PLoS One 16, no. 4: e0249833. 33844698 10.1371/journal.pone.0249833 PMC8041204

[jclp70093-bib-0050] Schafer, K. , G. A. Kennedy , A. Gallyer , and P. Resnik . 2021b. "Prediction of Suicide From Machine Learning and Traditional Theories: A Meta‐Analysis." PlosOne. 10.1371/journal.pone.0249833 PMC8041204 33844698

[jclp70093-bib-0034] Schafer, K. M. , E. Wilson , and T. Joiner . 2023. “Traumatic Brain Injury and Suicidality Among Military Veterans: The Mediating Role of Social Integration.” Journal of Affective Disorders 338: 414–421. 37364657 10.1016/j.jad.2023.06.047

[jclp70093-bib-0036] Schafer, K. M. , E. Wilson‐Lemoine , and T. Joiner . 2024. “Loneliness in Veterans: A Commonality Across Multiple Pathways Toward Suicidality.” Traumatology. 10.1037/trm0000509.

[jclp70093-bib-0039] Stack, S. 2000. “Suicide: A 15‐Year Review of the Sociological Literature Part Ii: Modernization and Social Integration Perspectives.” Suicide and Life‐Threatening Behavior 30, no. 2: 163–176. 10888056

[jclp70093-bib-0040] Stack, S. 2021. “Contributing Factors to Suicide: Political, Social, Cultural and Economic.” Preventive Medicine 152: 106498. 34538366 10.1016/j.ypmed.2021.106498

[jclp70093-bib-0041] Stack, S. , and L. Cao . 2020. “Social Integration and Indigenous Suicidality.” Supplement, Archives of Suicide Research 24, no. sup1: 86–101. 30734647 10.1080/13811118.2019.1572556

[jclp70093-bib-0042] Stack, S. , and J. Scourfield . 2015. “Recency of Divorce, Depression, and Suicide Risk.” Journal of Family Issues 36, no. 6: 695–715.

[jclp70093-bib-0043] Stevens, N. , and G. J. Westerhof . 2006. “Marriage, Social Integration, and Loneliness in the Second Half of Life: A Comparison of Dutch and German Men and Women.” Research on Aging 28, no. 6: 713–729.

[jclp70093-bib-0052] Tosti, G. 1898. Le Suicide: Étude de sociologie.

[jclp70093-bib-0044] Trudel‐Fitzgerald, C. , E. M. Poole , A. K. Sood , et al. 2019. “Social Integration, Marital Status, and Ovarian Cancer Risk: A 20‐Year Prospective Cohort Study.” Psychosomatic Medicine 81, no. 9: 833–840. 31592935 10.1097/PSY.0000000000000747 PMC6832885

[jclp70093-bib-0045] Tsai, A. C. , M. Lucas , and I. Kawachi . 2015. “Association Between Social Integration and Suicide Among Women in the United States.” JAMA Psychiatry 72, no. 10: 987–993. 26222043 10.1001/jamapsychiatry.2015.1002 PMC4598291

[jclp70093-bib-0046] Tsai, A. C. , M. Lucas , A. Sania , D. Kim , and I. Kawachi . 2014. “Social Integration and Suicide Mortality Among Men: 24‐Year Cohort Study of US Health Professionals.” Annals of Internal Medicine 161, no. 2: 85–95. 25023247 10.7326/M13-1291 PMC4137390

[jclp70093-bib-0047] Wang, L. , A. Seelig , S. M. Wadsworth , H. McMaster , J. E. Alcaraz , and N. F. Crum‐Cianflone . 2015. “Associations of Military Divorce With Mental, Behavioral, and Physical Health Outcomes.” BMC Psychiatry 15, no. 1: 128. 26087771 10.1186/s12888-015-0517-7 PMC4472413

[jclp70093-bib-0048] Weisenhorn, D. A. , L. M. Frey , J. van de Venne , and J. Cerel . 2017. “Suicide Exposure and Posttraumatic Stress Disorder: Is Marriage a Protective Factor for Veterans?” Journal of Child and Family Studies 26: 161–167.

